# Four New 2-(2-Phenylethyl)chromone Derivatives from Chinese Agarwood Produced via the Whole-Tree Agarwood-Inducing Technique

**DOI:** 10.3390/molecules21111433

**Published:** 2016-10-27

**Authors:** Yang-Yang Liu, De-Li Chen, Jian-He Wei, Jian Feng, Zheng Zhang, Yun Yang, Wei Zheng

**Affiliations:** 1 Institute of Medicinal Plant Development (National Engineering Laboratory for Breeding of Endangered Medicinal Materials), Chinese Academy of Medical Sciences & Peking Union Medical College, Beijing 100193, China; yyliu@implad.ac.cn (Y.-Y.L.); 18911570328@126.com (Z.Z.); yangyun43@aliyun.com (Y.Y.); 2 Hainan Branch of Institute of Medicinal Plant Development, Chinese Academy of Medicinal Sciences & Peking Union Medical College (Hainan Provincial Key Laboratory of Resources Conservation and Development of Southern Medicine), Haikou 570311, China; chendeli9999@163.com (D.-L.C.); jianfenghn@126.com (J.F.); wee000123@163.com (W.Z.)

**Keywords:** Chinese agarwood, 2-(2-phenylethyl)chromone derivatives, *Aquilaria sinensis*, whole-tree agarwood-inducing technique, anti-inflammatory

## Abstract

Four new 2-(2-phenylethyl)chromone derivatives (**1**–**4**) were isolated from the EtOH extract of Chinese agarwood produced via the whole-tree agarwood-inducing technique, coming from *Aquilaria sinensis* (Lour.) Spreng. (Thymelaeaceae). Their structures were elucidated by extensive spectroscopic methods, such as UV, IR, MS, 1D as well as 2D NMR. All of the isolates were then assessed for their anti-inflammatory activities on lipopolysaccharide (LPS)-induced nitric oxide (NO) production in RAW 264.7. Compound 1 exhibited significant inhibitory activity with an IC_50_ value of 4.6 μM.

## 1. Introduction

Agarwood or eaglewood (also known as *chen xiang* in China, *agar* in India, *oud* in the Middle East, *gaharu* in the South East Asia and *jinkoh* in Japan) is the resinous wood of the *Aquilaria* spp. trees. It is actually an angiosperm existing in the Thymelaeaceae family [1]. This cherished fragrant type of wood has for long been used as an incense, especially among the Buddhist, Hindu as well as for Islamic ceremonies [2]. Moreover, agarwood also plays a fundamental role in the traditional Chinese medicine due to of its medicinal value. It is considered to have sedative as well as carminative properties. Again, it has been used in relieving gastric problems, coughs, anti-emetic effects, rheumatism as well as high fever [2,3]. In China, the main source of agarwood is the Aquilaria sinensis, a large evergreen tree, distributed in the Hainan, Guangdong, Guangxi, and Fujian provinces.

Agarwood mainly forms in the wood tissues of wild or cultivated Aquilaria trees after wounding. This can mainly be caused by external factors like physical injury, insect gnawing, or microbial infection [4]. Usually, the tree takes several years to form the agarwood around the tissues wound. A lot of factors have led to the depletion of wild *Aquilaria* trees. These include agarwood’s immense value and rarity, indiscriminate cutting of trees, as well as over-harvesting. Our laboratory has therefore patented an effective method, which is the whole-tree agarwood inducing technique (Agar-Wit) [2,5,6], in China. In fact, it is currently being filed for international patent. In this technique, there were small holes deep into the xylem drilled into the main trunk of *Aquilaira* tree by use of an electric drill. The agarwood inducer was then slowly injected into the xylem tissues through a simple and cheap transfusion set to induce formation of high-quality agarwood in a shorter time compared to other conventional techniques.

**Figure 1:**
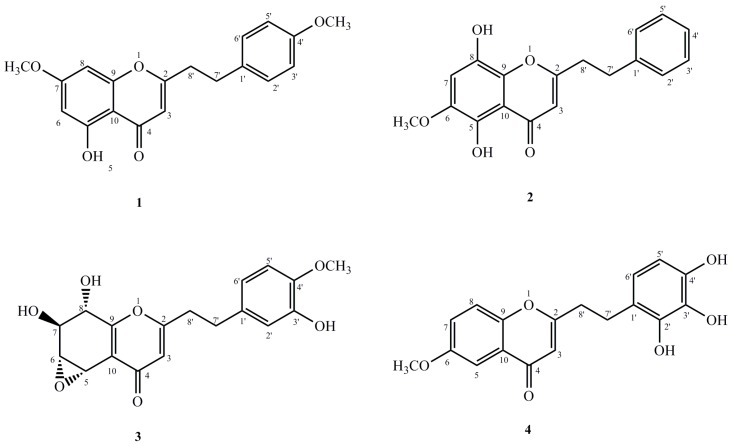
Structures of compounds **1**–**4**.

Previous phytochemical investigations on the Chinese agarwood revealed that chromone derivatives are among the main chemical components [7]. Some of these chemical components were also found to possess significant anti-inflammatory activity [8]. Nonetheless, chemical constituents of the Chinese agarwood were induced by the Agar-Wit technique from *A. sinensis* to contain just few reports [2]. As the inventor, it necessitated an investigation of the characteristic chromones of this agarwood. Our results gave out four new 2-(2-phenylethyl)chromone derivatives (**1**–**4**) (Figure 1) from the ethanol extract. This was also shown to be a moderate anti-inflammation activity. In this paper, the isolation and structural elucidation of these new compounds are described, merged with their inhibitory activities against LPS-induced NO production in macrophages.

## 2. Results and Discussion

**Figure 2:**
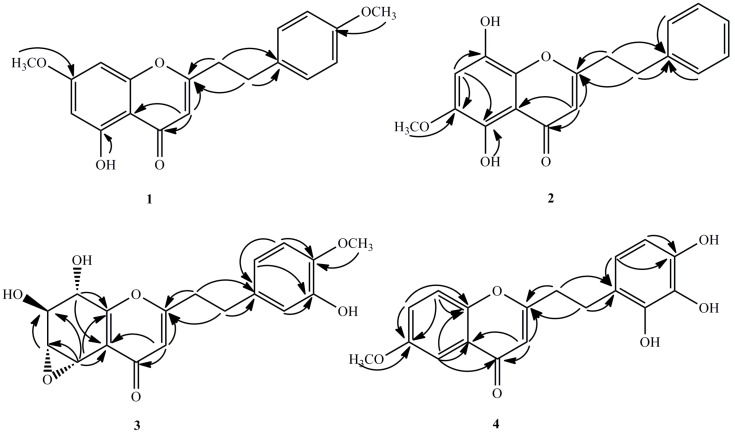
Key HMBC correlations of compounds **1**–**4.**

Compound **1** was isolated as pale yellow amorphous powder. Its molecular formula was determined to be C_19_H_18_O_5_ from the molecular ion peak at *m*/*z* 349.1023 [M + Na]^+^ (calcd for C_19_H_18_O_5_Na. 349.1052) from the HR-ESI-MS. Again, the IR spectrum indicated the presence of hydroxyl group (3415 cm^−1^) and α,β-unsaturated carbonyl group (1620 cm^−1^). The ^1^H-NMR spectroscopic data (Table 1) of **1** depicted the presence of two methoxyl groups at δ_H_ 3.90 and 3.88 (each 3H, s), one hydroxyl signal at δ_H_ 12.80, as well as two aromatic protons at δ_H_ 6.37 and 6.49. These were assigned to H-6 and H-8 respectively. In addition, a set of typical A_2_B_2_ coupling systems at δ_H_ 7.84 (2H, d, 9.0 Hz); δ_C_ 128.9, and δ_H_ 7.02; δ_C_ 114.8 (2H, d, 9.0 Hz), as well as four methylene protons at δ_H_ 2.35 (2H, d, 7.2 Hz, H-7′) and δ_H_ 2.80 (2H, d, 7.2 Hz, H-8′) were also observed from HSQC spectrum. Analysis of the ^13^C-NMR spectroscopic data (Table 2, see Supplementary Materials) showed that **1** had two methylene groups at δ_C_ 33.0 and 36.5, an α,β-unsaturated ketone at δ_C_ 108.0, 169.8 and 182.7, two methoxyl groups at δ_C_ 55.7 and 55.9, as well as other two aromatic rings in an AB and A_2_B_2_ patterns. Considering these, which suggested that compound **1** was a 2-(2-phenylethyl)chromone derivative with one hydroxyl as well as two methoxyl groups. Based on the HMBC spectroscopic experiment (Figure 2), the hydroxyl group was located at C-5 since the hydroxyl proton (δ_H_ 12.80) correlated with the carbon at C-5 (δ_C_ 149.8), C-10 (δ_C_ 110.8) as well as C-6 (δ_C_ 98.3). The methoxyl groups were attached to C-7 and C-4′, respectively. This is also on the basis of correlations from δ_H_ 3.90 to the aromatic carbon at δ_C_ 165.9, the δ_H_ 3.88 to the carbon at δ_C_ 157.0. Therefore, compound **1** was realised to be 5-hydroxyl-7-methoxy-2-[2-(4′-methoxyphenyl)ethyl]chromone.

Compound **2** was obtained as yellow amorphous powder. The molecular formula established as C_18_H_16_O_5_ by its HR-ESI-MS at *m*/*z* 335.0902 [M + Na]^+^ (calcd for C_18_H_16_O_5_Na, 335.0895). The IR spectrum also demonstrated absorption bands of hydroxyl group (3424 cm^−1^) as well as aromatic ring (1610, 1512 and 1455 cm^−1^). The UV spectrum further depicted the presence of an α,β-unsaturated carbonyl group out of the distinct absorption maximum at 242 and 322 nm. Moreover, the ^1^H-NMR spectrum (Table 1) also outlaid the presence of one methoxyl group at δ_H_ 3.93 (3H, s), one hydroxyl signal at δ_H_ 12.66, as well as one singlet aromatic protons at δ_H_ 6.86, and an A_2_B_2_X coupling system at δ_H_ 7.21(2H, t, 7.2 Hz), δ_C_ 128.4, δ_H_ 7.29(2H, d, 7.2 Hz), δ_C_ 128.9, and δ_H_ 7.27 (m), δ_C_ 123.3. The ^13^C-NMR spectrum (Table 2, see Supplementary Materials) of **2** illustrated signals for two methylene groups at δ_C_ 33.3 and 36.4, a trisubstituted double bond at δ_C_ 108.1 and 170.3, one methoxyl groups at δ_C_ 57.3, and a carbonyl group at δ_C_ 184.2. Basing on these findings, we realized that compound **2** was a 2-(2-phenylethyl)chromone derivative with two hydroxyl and one methoxyl groups which was then affirmed by the HMBC spectrum (Figure 2). In the HMBC spectrum, the correlations from methoxy signal (δ_H_ 3.93) to the carbon at δ_C_ 143.5 (C-6) and δ_H_ 6.86 (s, H-7) to δ_C_ 143.5 (C-6) indicated that the methoxy (δ_H_ 3.93) was located at C-6. Moreover, one hydroxyl group was linked to C-5 on the basis of the correlations between 5-OH (δ_H_ 12.66) and C-5 (δ_C_ 150.7). The other hydroxyl group was located at C-8, due to the downfield-shifted carbon at C-8 (δ_C_ 151.2) as well as the molecular formula above. Thus, the structure of compound **2** was assigned to be 5,8-dihydroxy-6-methoxy-2-(2-phenylethyl)chromone.

Compound **3** was obtained as a pale brown amorphous powder. The molecular formula established as C_18_H_18_O_7_ by its HR-ESI-MS at *m*/*z* 371.1103 [M + Na]^+^ (calcd for C_18_H_18_O_7_Na, 371.1107) the IR spectrum showed absorption bands of hydroxyl groups (3410, 3010 cm^−1^) and aromatic ring (1610, 1500 and 1425 cm^−1^). The ^1^H-NMR spectrum (Table 1) showed that the presence of one methoxyl group at δ_H_ 3.90 (3H, s), two methylene groups at δ_H_ 2.90, 2.98 (each 2H, t, *J* = 7.2 Hz), four oxygenated methine protons at δ_H_ 4.08 (dd, *J* = 10.2, 7.2 Hz), 4.32 (dd, *J* = 10.2, 4.8 Hz), 4.94 (d, *J* = 7.2 Hz), 5.02 (d, *J* = 4.8 Hz), and four aromatic protons at 6.62 (dd, *J* = 8.4, 1.8 Hz), 6.76 (d, *J* = 8.4 Hz), 6.74 (d, *J* = 1.8 Hz), 6.13 (s). The ^13^C-NMR spectrum (Table 2, see supporting information) displayed 18 carbon signals of two methylene groups (δ_C_ 32.4 and 35.6), a phenyl group (δ_C_ 119.8, 111.1, 146.0, 145.6, 114.5, and 132.3), a conjugated moiety (δ_C_ 114.1, 122.0, 157.0, 169.9, 179.8), four oxygenated methine carbons (δ_C_ 56.5, 61.9, 69.1, and 70.8), and one methoxy carbon (δ_C_ 56.2). From the ^1^H-NMR, ^1^H-^1^H COSY, and HSQC spectra, these four carbons were concluded to form a series of consecutive methines (δ_C_ 56.5, δ_H_ 5.02; δ_C_ 61.9, δ_H_ 4.32; δ_C_ 69.1, δ_H_ 4.08; δ_C_ 70.8, δ_H_ 4.94). In the HMBC spectrum (Figure 2), the methine proton at δ_H_ 5.02, which was located at one end of the consecutive methine, showed correlation peaks with the two olefinic carbons [δ_C_ 122.0 (C-10) and 157.0 (C-9)], whereas the methine proton at δ_H_ 4.94, which was located at the other end of the methine, correlated with the latter two olefinic carbons (δ_C_ 122.0 and 157.0). These correlations indicate that these methines form a part of a tetrasubstituted tetrahydrochromone moiety. The HMBC correlations from δ_H_ 3.90 and 6.76 to δ_C_ 146.0 indicated that the methoxy located at C-4′. The hydroxy linked at C-3′ (δ_C_ 145.6) because the δ_H_ 6.74 (H-2′) and δ_H_ 6.62 (H-6′) correlated with the carbon at C-3′. On the basis of ^1^H-NMR and ^13^C-NMR data, the structure of compound **3** is very similar to a reported compound named rel-(1a*R*,2*R*,3*R*,7b*S*)-1a,2,3,7b-Tetrahydro-2,3-dihydroxy-5-[2-(3-hydroxy-4-methoxy phenyl)ethyl]-7*H*-oxireno[*f*][1] benzopyran-7-one [9], except for two strongly downfield-shifted signals at δ_C_ 61.9 (C-6) and 56.5 (C-5) compared with the known one at δ_C_ 54.8 (C-6) and 49.5 (C-5), due to the relative configuration of epoxy group in this region, which was further confirmed by the NOESY correlations. In the NOESY spectrum, the correlations between H-5, H-6 and H-8 indicated the epoxy group was α-oriented. The *J*^3^-coupling constant (10.2 Hz) also supported an antiperiplanar relationship between H-6 and H-7. Therefore, the structure of compound **3** was assigned to be 5α,6α-epoxy-7β,8α,3′-trihydroxy-4′-methoxy-2-(2-phenylethyl)chromone.

Compound **4** was obtained as a pale brown amorphous powder. The molecular formula of compound **4** was determined to be C_18_H_16_O_6_ by HR-ESI-MS (*m*/*z* 351.0821 [M + Na]^+^, calc. for C_18_H_16_O_6_Na, 351.0845). The IR spectrum exhibited the presence of hydroxy group(s) at 3340 cm^−1^. The ^1^H-NMR spectrum (Table 1) showed the presence of one methoxyl group at δ_H_ 3.88 (3H, s), two methylene groups at δ_H_ 2.99, 2.89 (each 2H, t, *J* = 7.2 Hz), six aromatic protons at δ_H_ 6.13 (s), 7.55 (d, *J* = 3.0 Hz), 7.24, (dd, *J* = 9.0, 3.0 Hz), 7.37 (d, *J* = 9.0 Hz), 6.75 (d, *J* = 8.4 Hz) and 7.05 (d, *J* = 8.4 Hz). The ^13^C-NMR spectrum (Table 2, see supporting information) of **4** showed 18 carbon signals including one methoxy at δ_C_ 56.1, two methylene groups at δ_C_ 32.4 and 36.6, six methine at δ_C_ 109.7, 105.0, 123.8, 119.6, 115.7 and 129.6, and nine quaternary carbons at δ_C_ 168.6, 178.5, 157.0, 151.5, 124.4, 131.9, 151.2, 140.1 and 154.5. Based on the combined analyses of the IR, ^1^H-NMR, and ^13^C-NMR spectroscopic data, compound **4** was another 2-(2-phenylethyl)chromone derivative with one methoxyl group and three hydroxyl groups, which was further confirmed by HMBC correlations (Figure 2). In the HMBC spectrum, the correlations from the methoxy (δ_H_ 3.88) and H-7 (δ_H_ 7.24) to the carbon at δ_C_ 157.0, indicated that the methoxy (δ_H_ 3.88) was located on C-6 (δ_C_ 157.0), in which the integration value of H-7 was enhanced when the methoxy protons at δ_H_ 3.88 was irradiated. The positions of the hydroxyl groups were attached to C-2′/3′/4′, respectively on the basis of the downfield carbons at δ_C_ 151.2 (C-2′), 140.1 (C-3′), 154.5 (C-4′), together with the molecular formula above. Thus, the structure of **4** was identified as 6-methoxy-2-[2-(2′,3′,4′-trihydroxy)phenyl)ethyl]chromone.

Considering this medicinal herb as a therapeutical agent of analgesics and asthmatic, the isolated compounds **1**–**4** were studied for their anti-inflammatory activities on lipopolysaccharide (LPS)-induced nitric oxide (NO) production in RAW 264.7. The results showed that compound **1** showed significant inhibitory activities with IC_50_ value of 4.6 μM and compound **3** displayed moderate activity with IC_50_ value of 84 μM comparing of the positive drug control group aminoguanidine with IC_50_ value of 1.8 μM, while compounds **2** and **4** were inactive (Table 3).

## 3. Materials and Methods 

### 3.1. General Experimental Procedures

1D and 2D NMR spectra were obtained with a Bruker AV III 600NMR spectrometer (Bruker, Billerica, German) using TMS as the internal standard. HRESIMS spectra were performed on a LTQ-Obitrap XL spectrometer (Thermo Fisher Scientific, Boston, MA, USA). Optical rotations were obtained on a Perkin-Elmer 341 digital polarimeter (PerkinElmer, Norwalk, CT, USA). UV and IR spectra were recorded on Shimadzu UV2550 and FTIR-8400S spectrometers (Shimadzu, Kyoto, Japan), respectively. Semi-preparative LC was performed on a Lumtech K-1001 analytic LC (Beijing, China) which is equipped with two pumps of K-501, a UV detector of K-2600, as well as an YMC Pack C18 column (250 mm × 10 mm, i.d., 5 μM, YMC Co. Ltd., Kyoto, Japan) eluted with CH_3_CN–H_2_O (or MeOH–H_2_O) at a flow rate of 2 mL/min. ODS (12 nm–50 μm, YMC Co. Ltd., Kyoto, Japan), Sephadex LH-20 (Pharmacia, Uppsala, Sweden), as well as silica gel (100–200 and 300–400 mesh, Qingdao Marine Chemical plant, Qingdao, China) were utilized for column chromatography. Moreover, pre-coated silica gel GF_254_ plates (Zhi Fu Huang Wu Pilot Plant of Silica Gel Development, Yantai, China) were utilized for TLC (CH_2_Cl_2_:MeOH 100:1), the spots on TLC were detected by spraying with 5% H_2_SO_4_ in EtOH. All solvents utilized were of analytical grade (Beijing Chemical Works).

### 3.2. Plant Material

Agarwood which was induced by Agar-Wit from 7 years old *A. sinensis* tree was harvested about 18 months later, which was collected from Pingding Town, Huazhou City, Guangdong Province, China, in September 2014. The sample was identified by Prof. Jian-he Wei, Institute of Medicinal Plant Development, Chinese Academy of Medical Sciences and Peking Union Medical College, where a voucher specimen (No. 20140907) was deposited.

### 3.3. Extraction and Isolation

Dried and powdered Agarwood (10 kg) was also refluxed with 95% EtOH (50.0 L × 3) within conditions of reflux in yielding a semi-solid residue (850 g) which was made of the crude extract. The crude extracts were then dissolved successively with water (2 L), in the order, petroleum ether (MSO), dichloromethane (CH_2_Cl_2_), ethyl acetate (EtOAc) as well as *n*-butanol (*n*BuOH), in re-extracting water solution of the crude extract as well as obtaining different fractions.

The CH_2_Cl_2_ extract (86.5 g) was applied to silica gel (100–200 mesh) chromatographic column then it successively eluted with MSO–CH_2_Cl_2_ (*v*/*v*, 100:0–1:20, 3.0 L of each), CH_2_Cl_2_–MeOH (*v*/*v*, 100:0–0:100, 3.0 L of each) in providing twenty fractions (Fr. 1–Fr. 20). Fr. 1–9 mix with Fr. 10 (Fr. A) (10.5 g) was subjected to silica gel (200–300 mesh) chromatographic column as well as eluted with MSO–CH_2_Cl_2_ (*v*/*v*, 10:0–1:1, 0.5 L of each), CH_2_Cl_2_–MeOH (*v*/*v*, 100:0–0:100, 0.5 L of each) to get nine sub-fractions (Fr.A-1–Fr. A-9). Fr.A-3 (3.0 g) was subjected to further purified by semi-preparative liquid chromatography (LC) with CH_3_CN–H_2_O (*v*/*v*, 6:4) isolated to yield compound **1** (21.7 mg, *t*_R_ = 35.9 min).

Accordingly, the EtOAc extract (35.1 g) was also isolated to silica gel (100–200 mesh) chromatographic column and successively eluted with CH_2_Cl_2_–MeOH (*v*/*v*,100:0–0:100, 3.0 L of each) to give 7 fractions (Fr.A–Fr.G). Fr. A (8.1 g) was applied to silica gel (100–200 mesh) chromatographic column by eluted with MSO–CH_2_Cl_2_ (*v*/*v*, 1:1, 1:3, 1 L of each), CH_2_Cl_2_–MeOH (*v*/*v*, 100:0–0:100, 1.0 L of each) to provide six sub-fractions(Fr.A-1–Fr.A-6). Fr.A-3 (2.5 g) was chromatographed by ODS gel (3 × 40 cm) eluted with MeOH–H_2_O (*v*/*v*, 3:7, 1.0 L of each) to increase polarity to give eight sub-fractions (Fr.A-3-1–Fr.A-3-8). Compound **2** (7.3 mg, *t*_R_ = 22.2 min) and **4** (8.2 mg, *t*_R_ = 18.1 min) were separated out from the mixture of Fr.A-3-4-18~29 by semi-preparative LC with CH_3_CN–H_2_O (*v*/*v*, 50:50). Fr.A-3-2 (0.4 g) which was then purified by semi-preparative LC with MeOH–H_2_O (*v*/*v*, 44:56) isocratic to produce compound **3** (7.3 mg, *t*_R_ = 32.7 min).

The structures of compounds **1**–**4** were determined by UV, IR, ^1^H-NMR, ^13^C-NMR, ^1^H-^1^H COSY, HSQC, HMBC, NOESY and HR-ESI-MS.

*5-Hydroxy-7-methoxy-2-[2-(4′-methoxyphenyl)ethyl]chromone* (**1**). C_19_H_18_O_5_, pale yellow amorphous powder; m.p. 185–187 °C; UV λ_max_ (CHCl_3_) nm (log ε): 225 (4.14), 318 (3.66); IR (KBr) ν_max_ cm^−1^: 1030, 1220, 1275, 1365, 1480, 1620, 3415; HR-ESI-MS *m*/*z* 349.1023 [M + Na]^+^ (calcd. 349.1052); ^1^H-NMR spectra data, see Table 1; ^13^C-NMR spectrum data, see Table 2.

*5,8-Dihydroxy-6-methoxy-2-(2-phenylethyl)chromone* (**2**). C_18_H_16_O_5_, pale yellow amorphous powder; m.p. 124–127 °C; UV λ_max_ (CHCl_3_) nm (log ε): 242 (4.6), 322 (4.15); IR (KBr) ν_max_ cm^−1^ 1512, 1455, 1610, 3424; HR-ESI-MS *m*/*z* 335.0902 [M + Na]^+^ (calcd for C_18_H_16_O_5_Na, 335.0895); ^1^H-NMR spectra data, see Table 1; ^13^C-NMR spectrum data, see Table 2.

*5α,6α-Epoxy-7β,8α,3′-trihydroxy-4′-methoxy-2-(2-phenylethyl)chromone* (**3**). C_18_H_18_O_7_, pale brown amorphous powder, ${\left[\alpha \right]}_{D}^{20}$ −12.5 (*c* 0.10, MeOH); UV λ_max_ (MeOH) nm (log ε): 254 (4.02), 205 (4.45); IR (KBr) ν_max_ cm^−1^ 3410, 3010, 1660, 1610, 1500, 1425, 1265, 1240, 1120, 1100, 1015; HR-ESI-MS at *m*/*z* 371.1103 [M + Na]^+^ (calcd for C_18_H_18_O_7_Na, 371.1107); ^1^H-NMR spectra data, see Table 1; ^13^C-NMR spectrum data, see Table 2.

*6-Methoxy-2-[2-(2′,3′,4′-trihydroxy)phenyl)ethyl]chromone* (**4**). C_18_H_16_O_6_, pale brown amorphous powder; UV λ_max_ (MeOH) nm (log ε): 242 (4.56), 340 (3.75); IR (KBr) ν_max_ cm^−1^ 3340, 2545, 1610, 1512, 1405, 1310, 1284, 1140, 1020; HR-ESI-MS (*m*/*z* 351.0821 [M + Na]^+^, calc. for C_18_H_16_O_6_Na, 351.0845); ^1^H-NMR spectra data, see Table 1; ^13^C-NMR spectrum data, see Table 2.

### 3.4. Assay for Inhibitory Ability Against LPS-Induced NO Production in RAW 264.7 Macrophages

The in vitro anti-inflammatory activity was assessed through determining the nitrite concentration in the medium as well as the proliferation of RAW264.7 cells as illustrated in a previous study with some modify [10,11]. Shortly, the cells (10^5^ cells/well) were co-incubated with drugs (Compounds **1**–**4** and Aminoguanidine) as well as LPS (1 μg/mL) for 24 h at 37 °C. The tested samples were dissolved in DMSO, and then diluted with DMEM to make the final DMSO concentration of 0.1%. After that, the cells were coincubated with fresh medium (150 μL/well) and then treated with LPS (200 ng/mL), and the tested compounds at various concentrations (0.2–50.0 μM) for 24 h. Griess reagent was used to determine the NO production by detecting the nitrite in the culture supernatant. In short, 100 μL of the culture supernatant was reacted with an equal volume of Griess reagent and vibrated for 10 min at room temperature. The amount of NO was assessed by finding the nitrite concentration in the cultured RAW 264.7 macrophage supernatants with Griess reagent. Aliquots of supernatants (100 μL) were also incubated, in-sequence, with 50 μL of 1% sulfanilamide and 50 μL of 0.1% naphthylethylenediamine in 2.5% phosphoric acid solution. From this, the absorbance was recorded on a micro-plate reader at a wavelength of 570 nm. The results were expressed as IC_50_ values which were calculated using the CalcuSyn program and expressed as the means with SD of three independent experiments.

## Figures and Tables

**Table 1:** ^1^H-NMR (600 MHz) assignments of compounds **1**–**4** (CDCl_3_).

Attribution	δ_H_ (*J* in Hz)			
	1	2	3	4
3	6.59, s	6.04, s	6.13, s	6.13, s
5	-	-	5.02, d (4.8)	7.55, d (3.0)
6	6.37, d (2.4)	-	4.32, dd (10.2, 4.8)	-
7	-	6.86, s	4.08, dd (10.2, 7.2)	7.24, dd (9.0, 3.0)
8	6.49, d (2.4)	-	4.94, d (7.2)	7.37, d, (9.0)
2′	7.84, d (9.0)	7.29, d (7.2)	6.74, d (1.8)	-
3′	7.02, d (9.0)	7.21, t (7.2)	-	-
4′	-	7.27, m	-	-
5′	7.02, d (9.0)	7.21, t (7.2)	6.76, d (8.4)	6.75, d (8.4)
6′	7.84, d (9.0)	7.29, d (7.2)	6.62, dd (8.4, 1.8)	7.05, d (8.4)
7′	2.35, t (7.2)	3.05, m	2.98, t (7.2)	2.99, t (7.2)
8′	2.80, t (7.2)	2.95, m	2.90, t (7.2)	2.89, t (7.2)
6-OCH_3_	-	3.93, s	-	3.88, s
7-OCH_3_	3.90, s	-	-	-
4′-OCH_3_	3.88, s	-	3.90, s	-
5-OH	12.80, s	12.66, s	-	-

**Table 2:** ^13^C-NMR (150 MHz) assignments of compounds **1**–**4** (CDCl_3_).

Attribution	δ_C_			
	1	2	3	4
2	169.8	170.3	169.9	168.6
3	108.0	108.1	114.1	109.7
4	182.7	184.2	179.8	178.5
5	149.8	150.7	56.5	105.0
6	98.3	143.5	61.9	157.0
7	165.9	105.9	69.1	123.8
8	92.9	151.2	70.8	119.6
9	150.6	149.8	157.0	151.5
10	110.8	111.1	122.0	124.4
1′	140.0	139.9	132.3	131.9
2′	128.9	128.9	114.5	151.2
3′	114.8	128.4	145.6	140.1
4′	157.0	123.3	146.0	154.5
5′	114.8	128.4	111.1	115.7
6′	128.9	128.9	119.8	129.6
7′	33.0	33.3	32.4	32.4
8′	36.5	36.4	35.6	36.6
6-OCH_3_	-	57.3	-	56.1
7-OCH_3_	55.9	-		-
4′-OCH_3_	55.7	-	56.2	-

**Table 3:** Inhibitory activity of compounds on lipopolysaccharide (LPS)-induced nitric oxide (NO) production in RAW 264.7 macrophages.

Compounds	IC_50_^a^ (μM)
**1**	4.6 ± 0.1
**2**	NA
**3**	84 ± 2
**4**	NA
Aminoguanidine ^b^	1.8 ± 0.2 μM

^a^ Value present mean ± SD of triplicate experiments. ^b^ Positive control substance. NA: No Activity.

## Supplementary Materials

The following are available online at: http://www.mdpi.com/1420-3049/21/11/1433/s1. The NMR (1D and 2D) of compounds **1**–**4** are available as Supplementary Materials.
