# Supplementary material for: Four New 2-(2-Phenylethyl)chromone Derivatives from Chinese Agarwood Produced via the Whole-Tree Agarwood-Inducing Technique
Source: Molecules. 2016 Oct 27;21(11):1433. doi: 10.3390/molecules21111433 (PMC6274528; doi:10.3390/molecules21111433)
Supplement: Supplementary file 1 [file molecules-21-01433-s001.pdf]

Supplementary Materials: Four New 2-(2-Phenylethyl) Chromone Derivatives from Chinese Agarwood Produced via the Whole-Tree Agarwood-Inducing Technique

Yang-Yang Liu, De-Li Chen, Jian-He Wei, Jian Feng, Zheng Zhang, Yun Yang and Wei Zheng

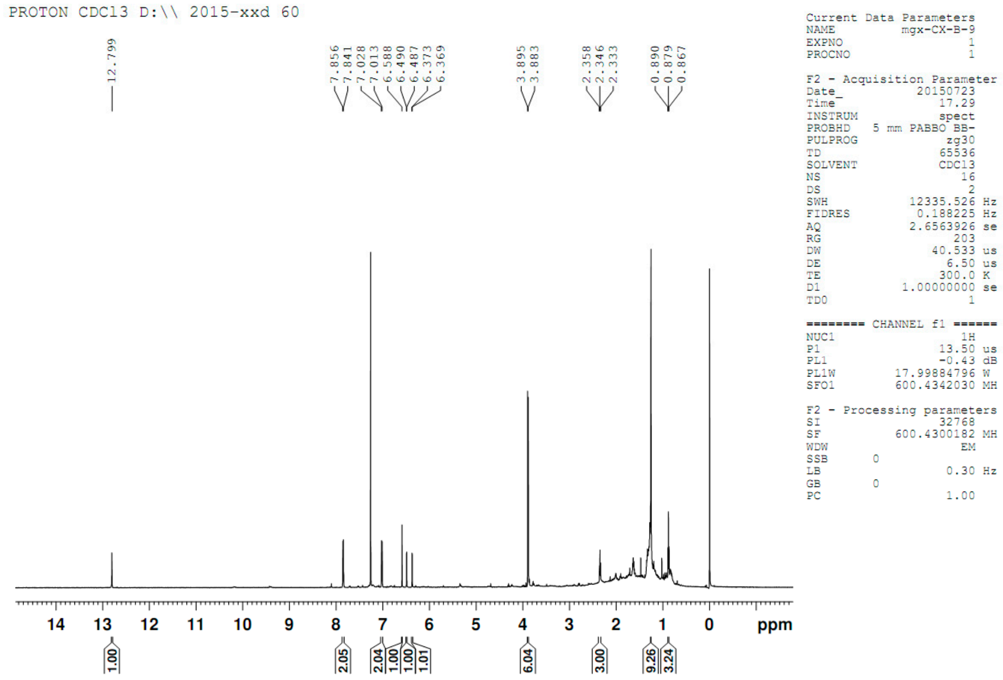

Figure S1. ^{1}H-NMR (600 MHz, $CDCl_3$) spectrum of the new compound **1**.

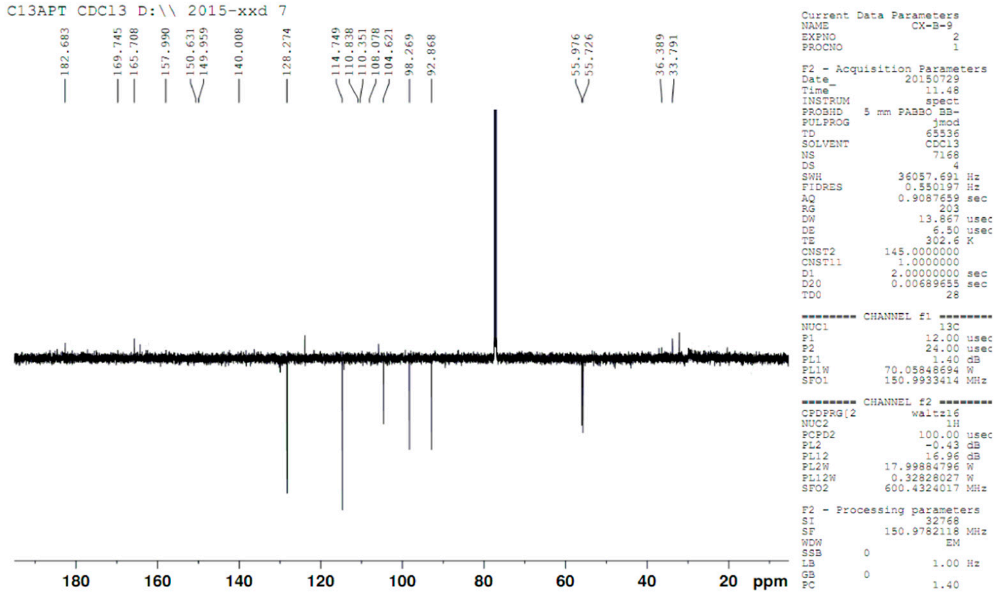

Figure S2. ^{13}C-APT (150 MHz, $CDCl_3$) spectrum of the new compound **1**.

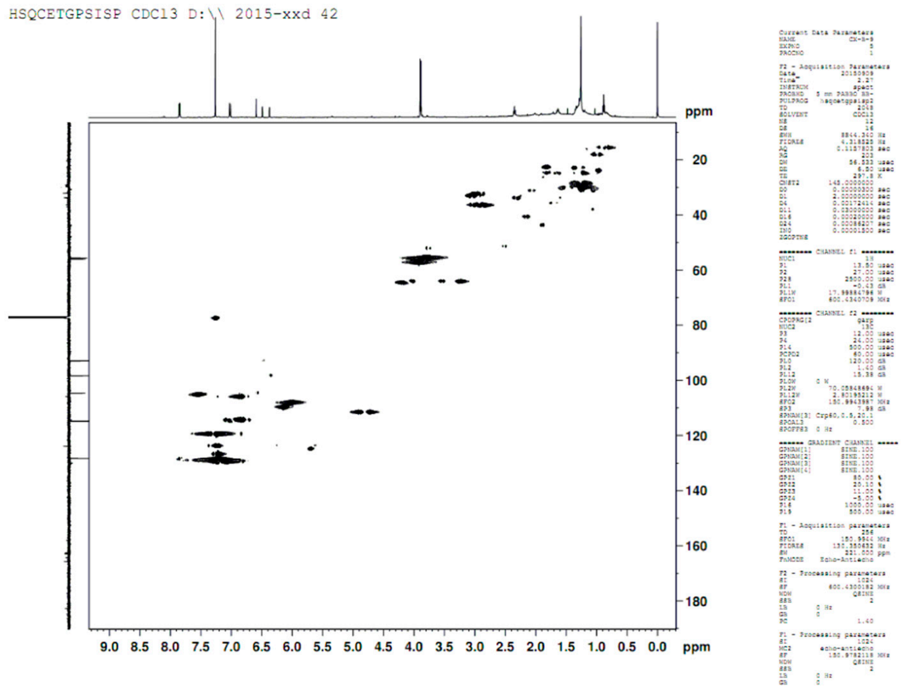

Figure S3. HSQC (600 MHz, $CDCl_3$) spectrum of the new compound **1**.

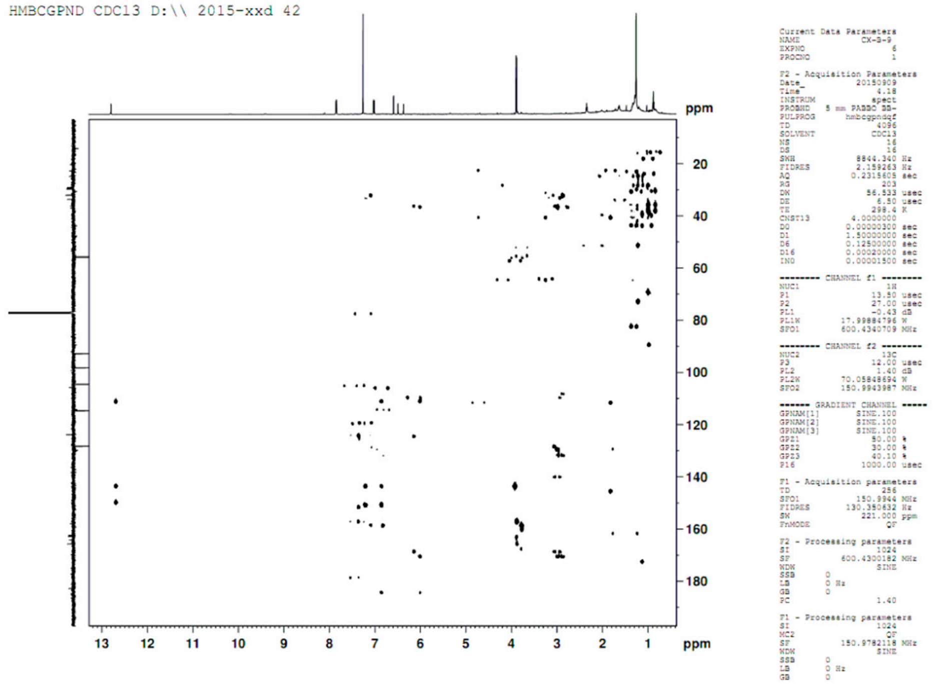

Figure S4. HMBC (600 MHz, $CDCl_3$) spectrum of the new compound **1**.

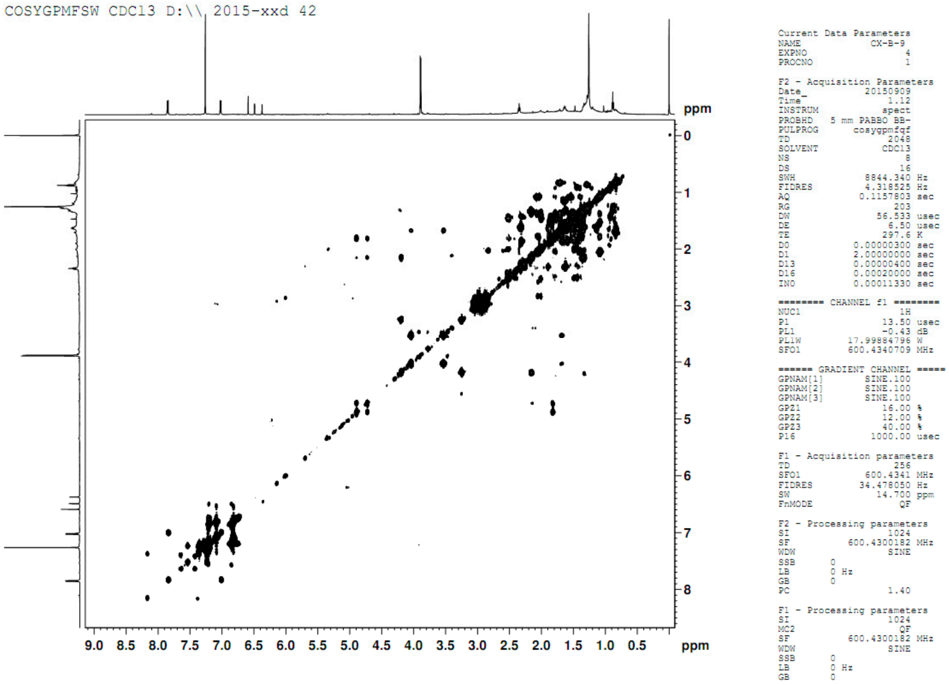

Figure S5. ^1H-^1H COSY (600 MHz, $CDCl_3$) spectrum of the new compound **1**.

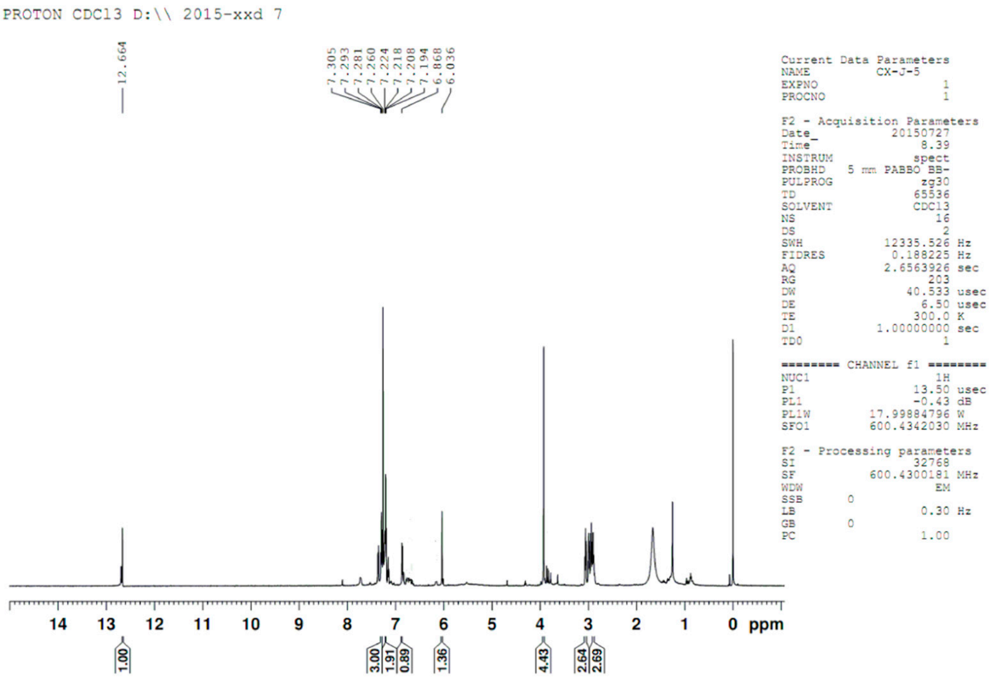

Figure S6. ^{1}H-NMR (600 MHz, $CDCl_3$) spectrum of the new compound **2**.

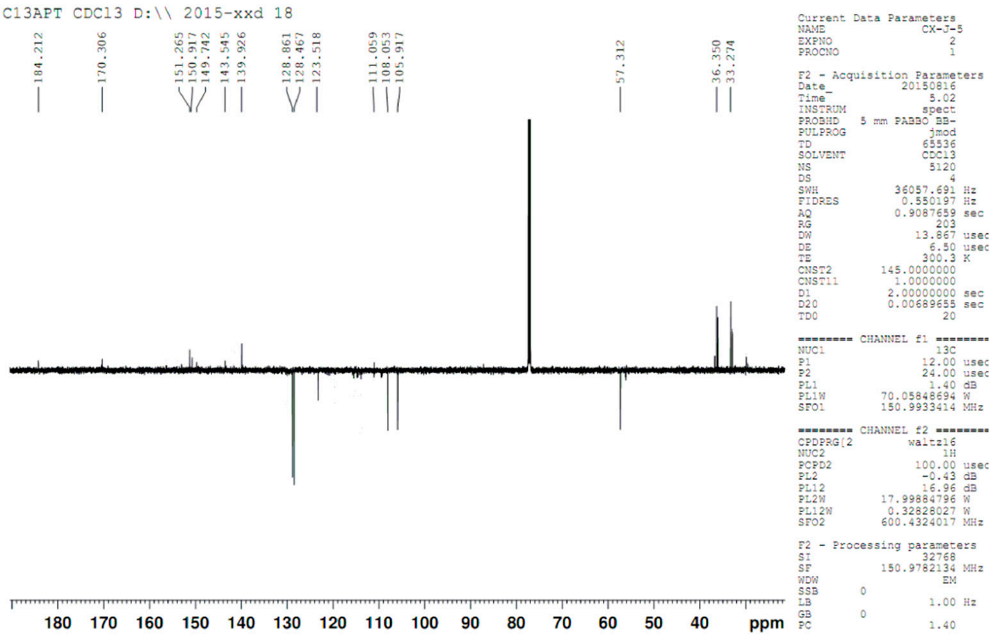

Figure S7. ^{13}C-APT (150 MHz, $CDCl_3$) spectrum of the new compound **2**.

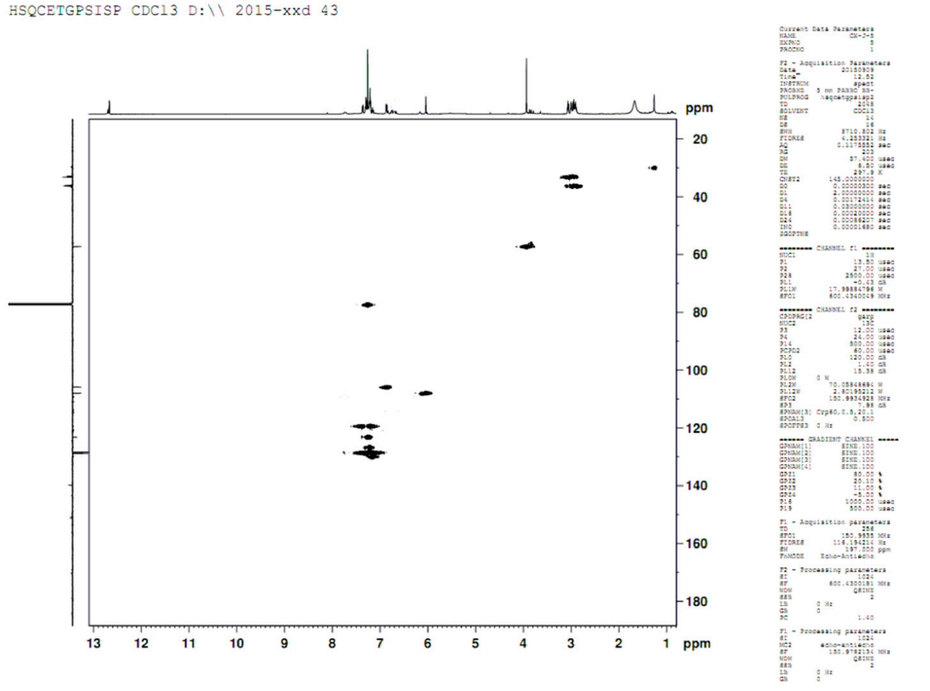

Figure S8. HSQC (600 MHz, $CDCl_3$) spectrum of the new compound **2**.

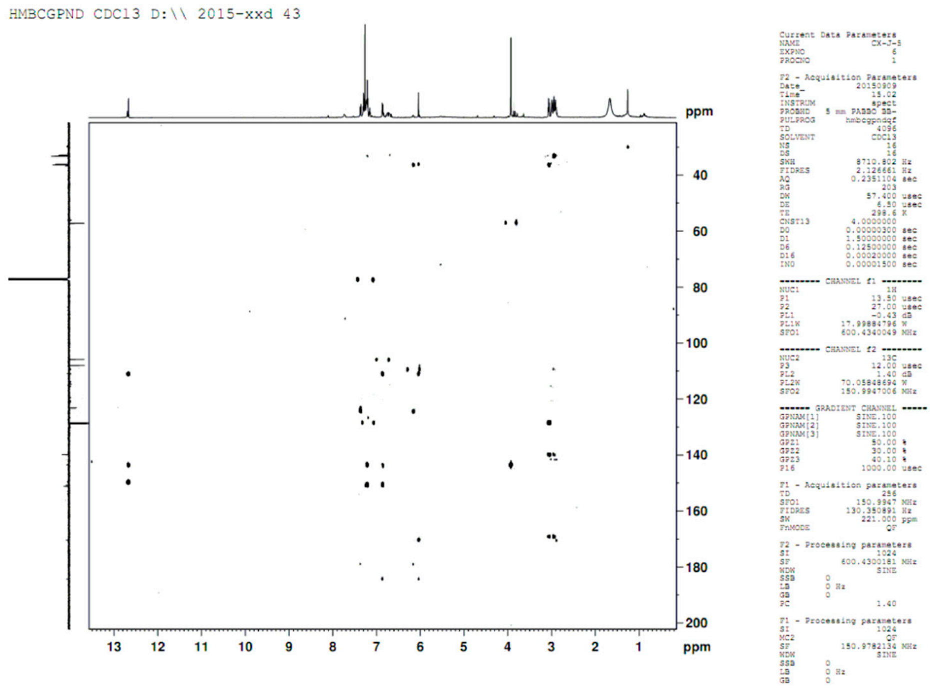

Figure S9. HMBC (600 MHz, $CDCl_3$) spectrum of the new compound **2**.

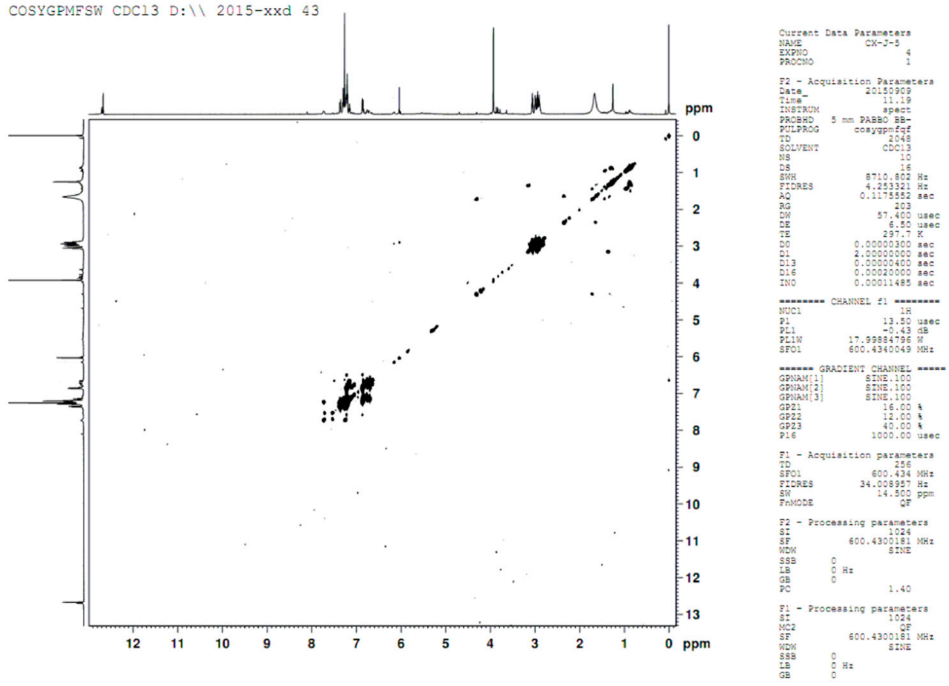

Figure S10. ^{1}H-^{1}H COSY (600 MHz, $CDCl_3$) spectrum of the new compound **2**.

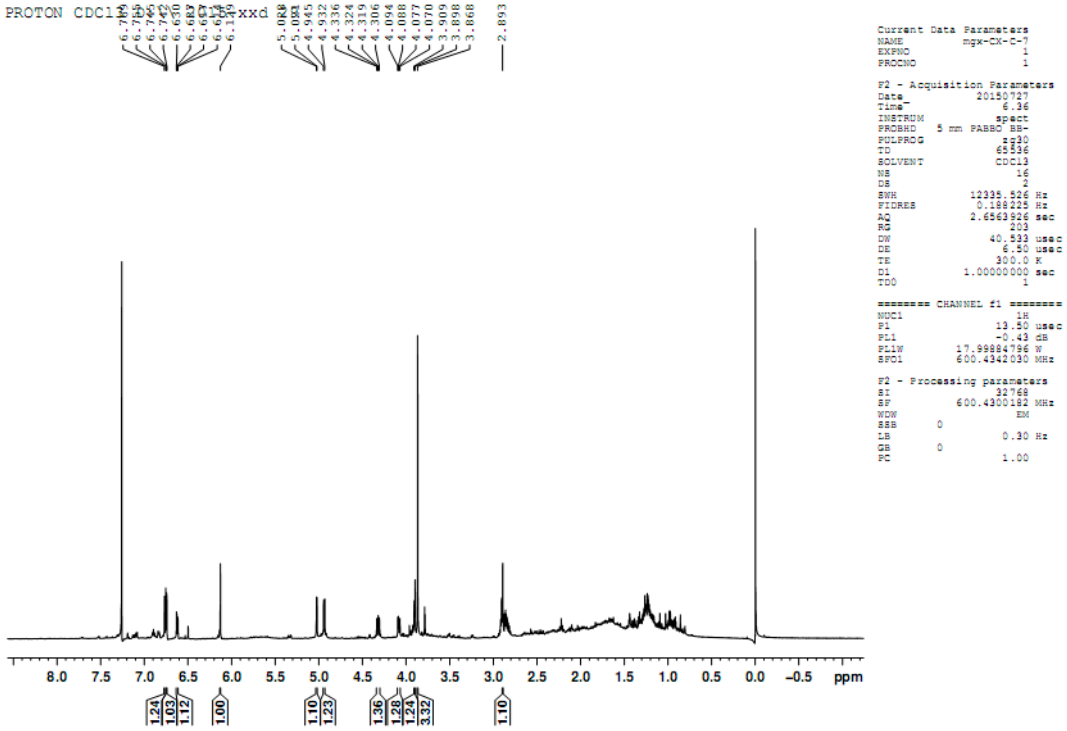

Figure S11. ^{1}H-NMR (600 MHz, $CDCl_3$) Spectrum of Compound **3**.

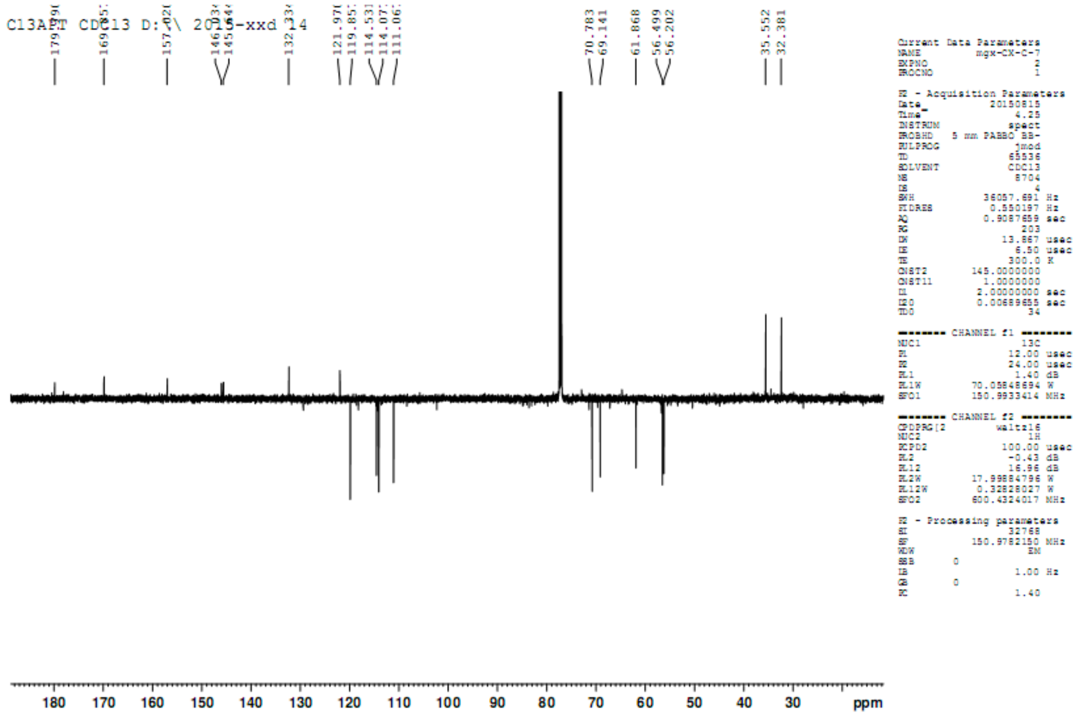

Figure S12. ^{13}C-APT (150 MHz, $CDCl_3$) Spectrum of Compound **3**.

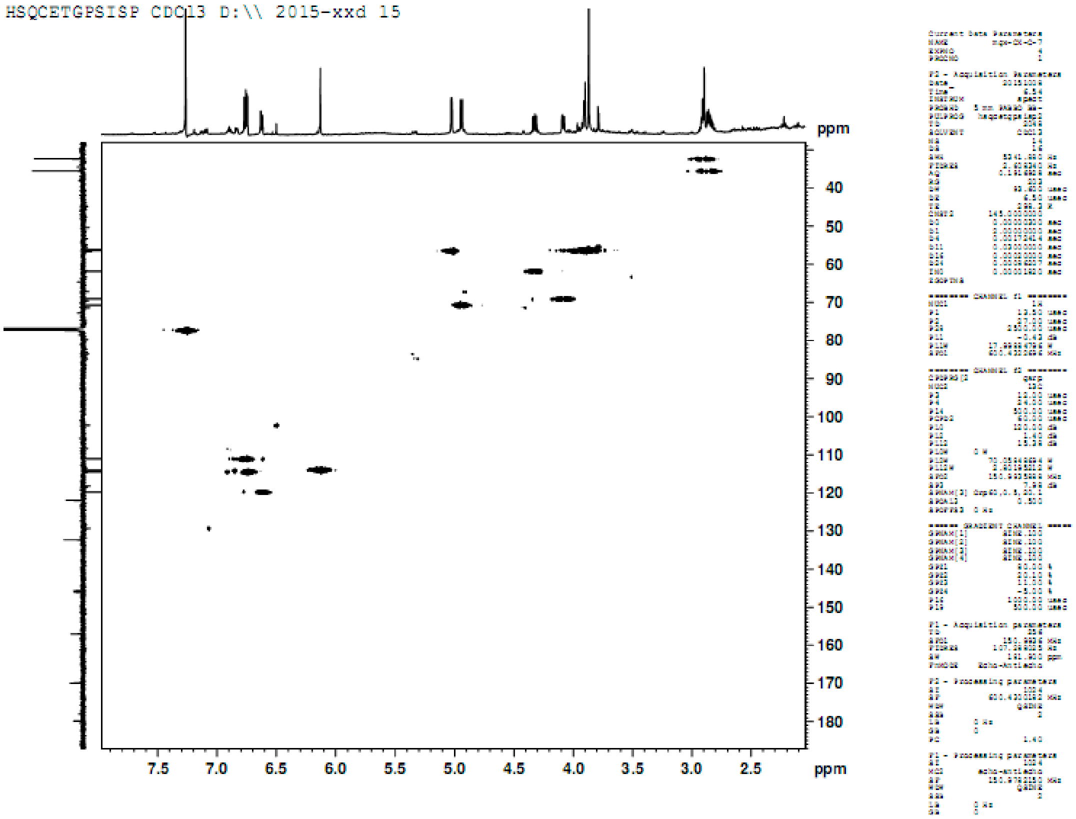

Figure S13. HSQC (600 MHz, $CDCl_3$) spectrum of the new compound **3**.

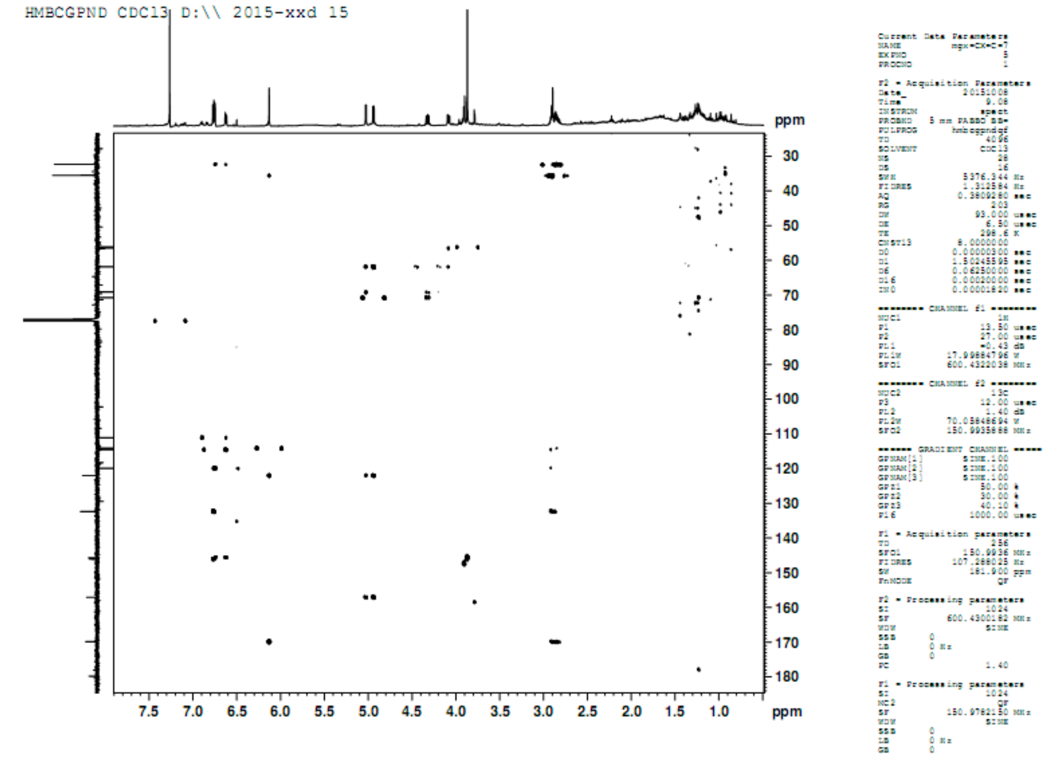

Figure S14. HMBC (600 MHz, $CDCl_3$) spectrum of the new compound **3**.

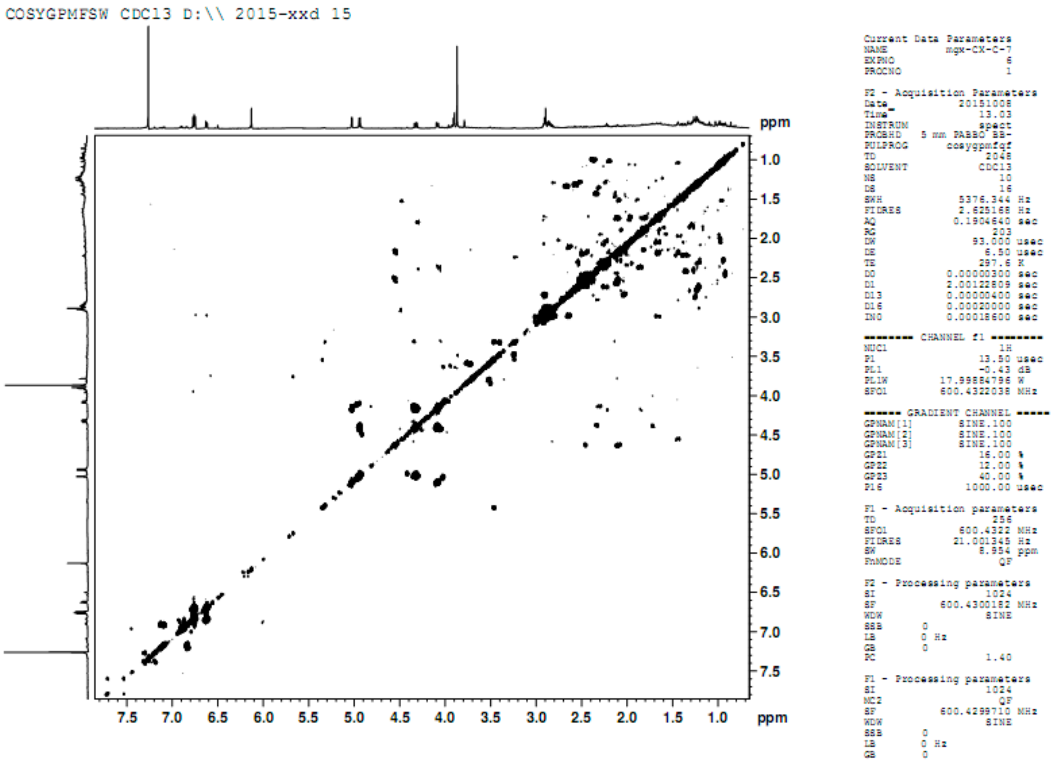

Figure S15. ^1H-^1H COSY (600 MHz, $CDCl_3$) spectrum of the new compound **3**.

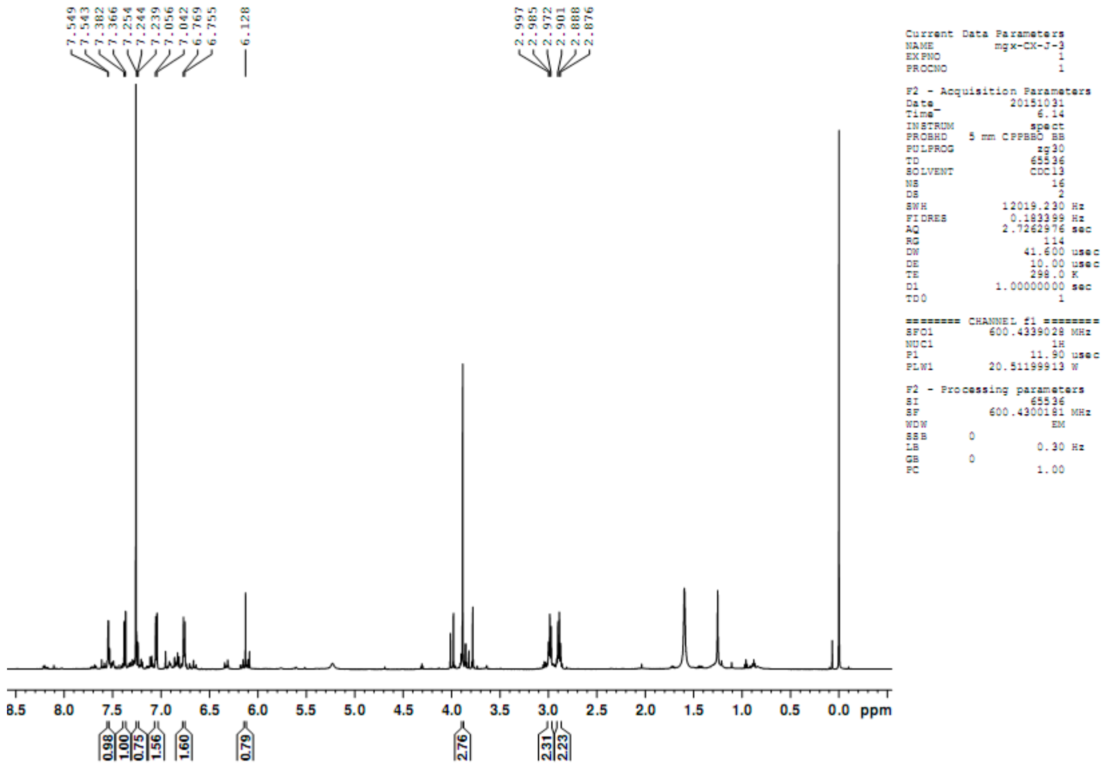

Figure S16. ^{1}H-NMR (600 MHz, $CDCl_3$) spectrum of the new compound **4**.

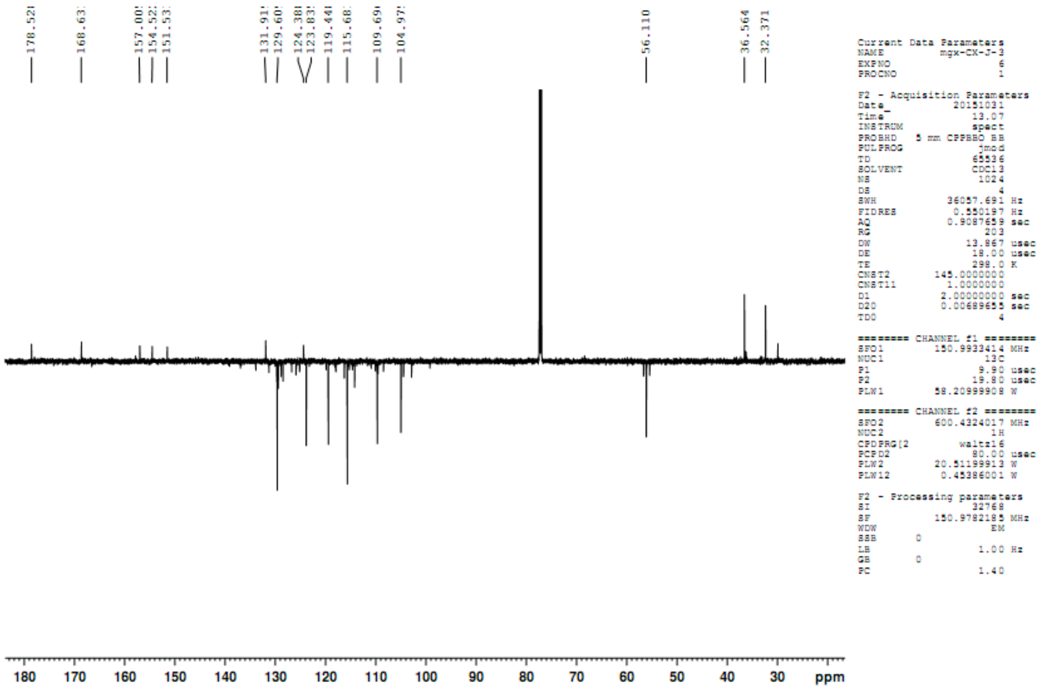

Figure S17. ^{13}C-APT (150 MHz, $CDCl_3$) spectrum of the new compound **4**.

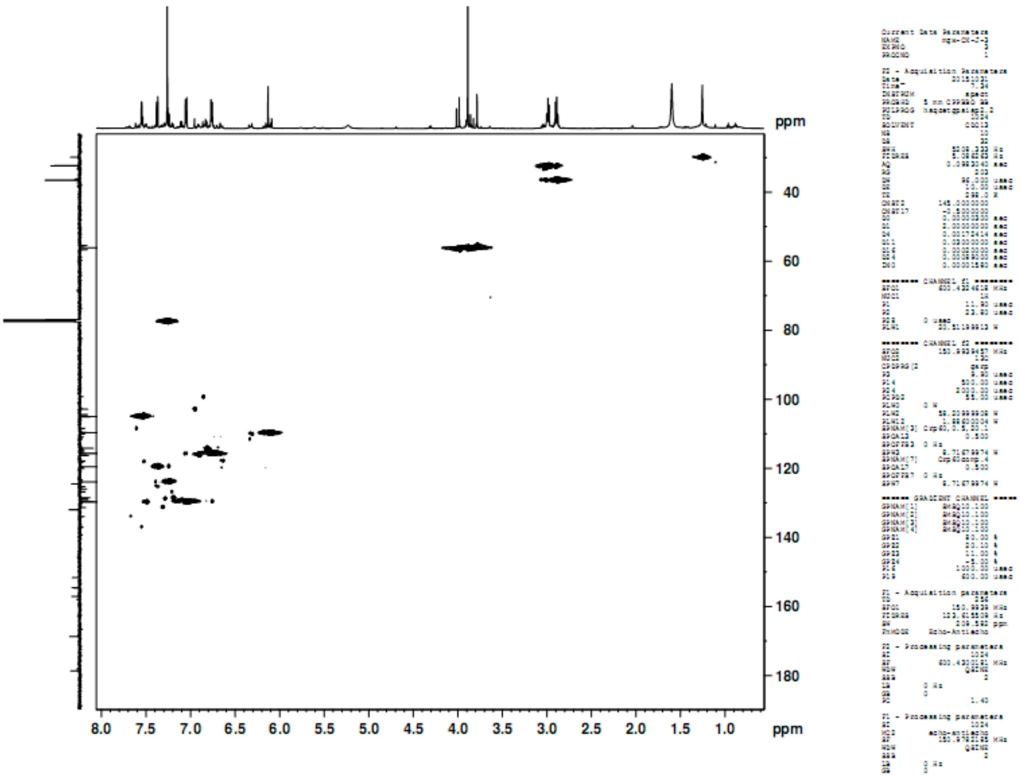

Figure S18. HSQC (600 MHz, $CDCl_3$) spectrum of the new compound **4**.

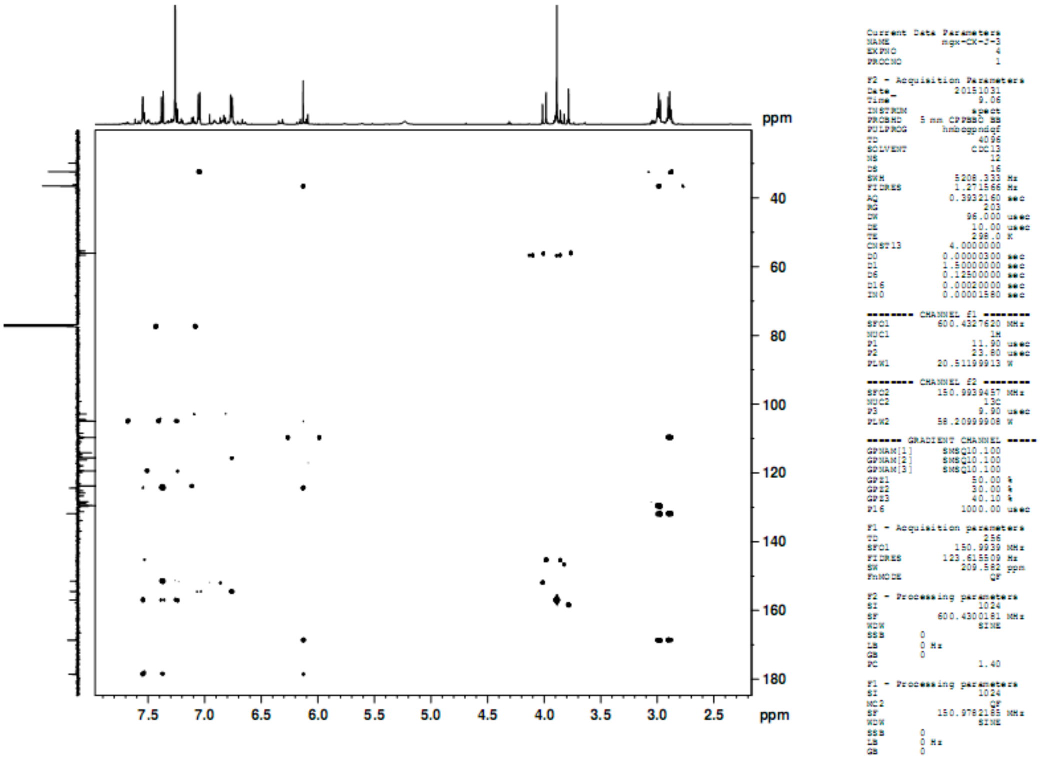

Figure S19. HMBC (600 MHz, $CDCl_3$) spectrum of the new compound **4**.

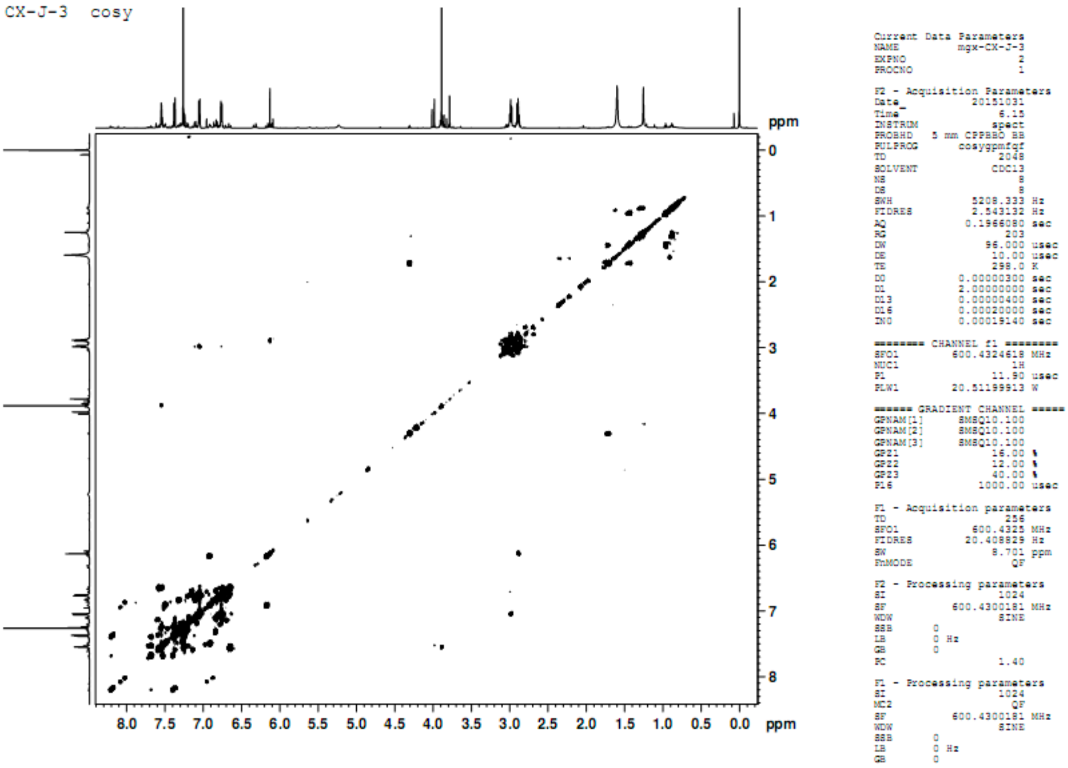

Figure S20. ^{1}H-^{1}H COSY (600 MHz, $CDCl_3$) spectrum of the new compound **4**.
